# Thermoauxetic Behavior of Composite Structures

**DOI:** 10.3390/ma11020294

**Published:** 2018-02-13

**Authors:** Hubert Jopek, Tomasz Stręk

**Affiliations:** Institute of Applied Mechanics, Poznan University of Technology, ul. Jana Pawła II 24, 60-965 Poznan, Poland; tomasz.strek@put.poznan.pl

**Keywords:** auxetic, thermoauxetic, thermal auxeticity, anti-tetrachiral, re-entrant honeycomb, tunable material properties

## Abstract

This paper presents a study of new two-dimensional composite structures with respect to their thermomechanical properties. The investigated structures are based on very well-known auxetic geometries—i.e., the anti-tetrachiral and re-entrant honeycomb—modified by additional linking elements, material which is highly sensitive to changes of temperature. The study shows that temperature can be used as a control parameter to tune the value of the effective Poisson’s ratio, which allows, in turn, changing its value from positive to negative, according to the temperature applied. The study shows that such thermoauxetic behavior applies both to composites with voids and those completely filled with material.

## 1. Introduction

It is very well known that mechanical properties of materials depend on temperature. In particular, temperature change can drastically influence the values of materials’ elastic properties, i.e., the Young’s modulus and the Poisson’s ratio [1,2]. This is particularly interesting in the case of auxetic materials. The negative Poisson’s ratio characterizing auxetic materials has been extensively studied for more than thirty years. Since first works by Gibson [3], Lakes [4], Wojciechowski [5] and Evans [6], it has been discovered that auxeticity can be obtained from specific geometry of material microstructure and, consequently, many such geometries have been proposed. Some of the most popular geometries are: chiral and antichiral [7,8,9], re-entrant [10,11], double arrowhead [12,13] and rotating polygons [14]. Auxetic behavior could be also the result of precisely designed cuts, which have been shown by Francesconi et al. [15] and Javid et al. [16] All these geometries are usually used to build cellular structure of materials. Studies of these structures have been performed both experimentally and theoretically, using numerical methods, e.g., a review of the manufacture, mechanical properties of auxetic foams was published by Critchley et al. [17].

A very important group of materials are composites consisting of two or more constituents. Composites in which at least one phase is auxetic have also been considered by some authors. For instance, Strek et al. studied the behavior of torsioned composite beams in which one phase is characterized by negative Poisson’s ratio [18,19]; Lim investigated laminates with auxetic phase [20,21] as well as other systems characterized by negative Poisson’s ratio [22]. Jopek studied a sandwich panel reinforced with unidirectional auxetic fibers [23]. The composite’s material characteristics depend both on mechanical properties of each constituent and topological arrangement of each phase. These effective properties of composites can be very different from the properties of each constituent.

Further, there are composites whose one or more material phases create auxetic structure. Milton [24] investigated hexagonal composites characterized by the Poisson’s ratio close to −1. Evans et al. [25] analyzed re-entrant network embedded in another matrix material. Experimental studies on.

Sigmund and Bendsøe [26,27] showed in particular that it was possible to perform an optimization procedure in order to design a composite structure characterized by specific effective material properties. In case of two-phase composites composed of elastically isotropic constituents with bulk and shear moduli, Hill [28] provided first bounds on the range of effective bulk and shear moduli based on the Voigt and Reuss estimates. Hashin and Shtrikman [29] improved these bounds for three-dimensional composites and well-ordered constituents (phase with larger shear modulus has also larger bulk modulus). In anisotropic solids (e.g., single crystals, honeycombs and fibrous composites), physical properties, including Poisson’s ratio and elastic moduli, depended on direction and could have positive or negative values of arbitrarily large size. Strek et al. [30,31] proposed optimized topologies of a composite built out of two materials characterized by positive Poisson’s ratio whose effective Poisson’s ratio was negative. Czarnecki et al. presented models of auxetic materials resulting from optimal distribution of Young’s modulus within the composite material volume [32,33]. Long et al. maximized the effective Young’s modulus of a composite with auxetic inclusions [34]. Pozniak et al. [35] investigated composites with elliptic inclusions using the finite element method and showed that one could tailor a material of practically arbitrary elastic parameters.

Voids occurring in the internal structure are a common feature of auxetics, including most composites characterized by the negative effective Poisson’s ratio. Still, composites were proposed whose whole volume was filled with material and which yet exhibited auxetic behavior. Recently, new research was published in which the value of effective Poisson’s ratio could be tuned from positive to negative. Grima et al. [36] proposed metamaterial in which the value of the Poisson’s ratio was controlled by an external magnetic field. Jopek and Strek [37,38] presented a composite in which the value of the Poisson’s ratio switched from positive to negative as a function of temperature. Similar results were obtained by Li at al. [39,40] in their studies of the bimetallic re-entrant structure.

In this paper, new composite structures are proposed based on very well-known auxetic geometries, i.e., anti-tetrachiral and re-entrant honeycomb. Modified structures are built with two constituent materials—each of them is characterized by different temperature-dependent elastic properties. Mechanical properties of the resultant composites are also influenced by temperature. The article focuses in particular on the effective Poisson’s ratio and the possibility of changing its value from positive to negative depending on the temperature applied (thermoauxeticity). FEM method is used to perform numerical calculations.

## 2. Materials and Methods

The equilibrium equation expressed in terms of stresses can be written in a compact notation

(1) −∇ · σ=F,

where F denotes the volume force (body force), σ is the stress tensor. In a linearly elastic material, stress tensor is σ=C:ε, where C is the fourth-order stiffness tensor and the strain-displacement relation for displacement is $\varepsilon =\frac{1}{2}\left(\nabla u+{\left(\nabla u\right)}^{T}\right)$. Generally, for linear isotropic material, only two constants are needed to describe the elastic behavior of the material: The Young’s modulus and the Poisson’s ratio, or the shear modulus (G) and bulk modulus (K), where G=E/(2(1+ν)) and K=E/(3(1−2ν)), or two Lamé constants. The Poisson’s ratio of an isotropic, linearly elastic material can neither be less than −1.0 nor greater than 0.5. Hooke’s law (solid stress-strain relationship) for the linear isotropic material is:

(2) $$\sigma =2\mu \varepsilon +\lambda \left(\nabla \;\cdot \;u\right)\delta_{ij},$$

where u is the displacement field, $\delta_{ij}$ is Kronecker’s delta and λ and μ are Lamé constants:

(3) $$\mu =G=\frac{E}{2\left(1+\nu \right)},\;\lambda =\frac{E\;\nu }{\left(1-2\nu \right)\left(1+\nu \right)}.$$

Substituting the stress-strain and strain-displacement relationship in the Navier’s Equation (1) results in the Navier’s equation expressed in the displacement. Neglecting body forces Navier’s equation is described as follows:

(4) $$\mu \nabla^{2}u+\left(\lambda +\mu \right)\nabla \left(\nabla \;\cdot \;u\right)=0.$$

The Navier’s equation is desired formulation for the displacement problem and the system represents three equations (in three-dimensional problems) for the three unknown displacement components.

However, many problems in elasticity may be treated satisfactorily by a two-dimensional plane theory of elasticity. There are two general types of problems involved in this plane analysis (plane stress or plane strain). These types will be defined by setting down certain restrictions and assumptions on the stress and displacement fields. For the simplicity of writing expressions, it is assumed that $x=\left[x_{1},x_{2},x_{3}\right]=\left[x,y,z\right]$ and $u=\left[u_{x},u_{y},u_{z}\right]$.

Plane stress is defined to be a state of stress in which the normal stress $\sigma_{z}$ and the shear stresses $\sigma_{xz}$ and $\sigma_{yz}$, directed perpendicular to the *xy*-plane are assumed to be zero. The geometry of the body is essentially that of a plate with one dimension much smaller than the others. The loads are applied uniformly over the thickness of the plate and act in the plane of the plate.

For isotropic materials and assuming $\sigma_{z}=\tau_{xz}=\;\tau_{yz}=0$ and $\gamma_{xz}=\gamma_{yz}=0$ yields σ=C:ε, where stiffness tensor is expressed as:

(5) $$C=\frac{E}{1-\nu^{2}}\left[\begin{array}{ccc} 1 & \nu & 0\\ \nu & 1 & 0\\ 0 & 0 & \frac{1-\nu }{2} \end{array}\right].$$

The basic partial differential equations for plane stress without body and inertia forces are:

(6) $$G\left(\frac{\partial^{2}u_{x}}{\partial x^{2}}+\frac{\partial^{2}u_{x}}{\partial y^{2}}\right)+\;\frac{1+\nu }{1-\nu }G\frac{\partial }{\partial x}\;\left(\frac{\partial u_{x}}{\partial x}+\frac{\partial u_{y}}{\partial y}\right)=0,\;G\left(\frac{\partial^{2}u_{y}}{\partial x^{2}}+\frac{\partial^{2}u_{y}}{\partial y^{2}}\right)+\;\frac{1+\nu }{1-\nu }G\frac{\partial }{\partial y}\;\left(\frac{\partial u_{x}}{\partial x}+\frac{\partial u_{y}}{\partial y}\right)=0.$$

Plane strain is defined to be a state of strain in which the strain normal to the *xy*-plane $\varepsilon_{z}$ and the shear strain $\gamma_{xz}$ and $\gamma_{yz}$ are assumed to be zero. In plane strain, the dimension of the structure in one direction (say the *z*-direction) is very large in comparison with the dimensions of the structure in the other two directions. The loads are uniformly distributed with respect to the large dimension and act perpendicularly to it.

For isotropic materials and assuming $\varepsilon_{z}=\gamma_{xz}=\;\gamma_{yz}=0$ and $\gamma_{xz}=\gamma_{yz}=0$ yields σ=C:ε, where stiffness tensor is expressed as:

(7) $$C=\frac{E}{\left(1+\nu \right)\left(1-2\nu \right)}\left[\begin{array}{ccc} 1-\nu & \nu & 0\\ \nu & 1-\nu & 0\\ 0 & 0 & \frac{1-2\nu }{2} \end{array}\right],$$

with

(8) $$\sigma_{z}=\frac{E}{1+\nu }\frac{\nu }{1-2\nu }\left(\varepsilon_{x}+\varepsilon_{y}\right)$$

The basic partial differential equations for plane strain are:

(9) $$G\left(\frac{\partial^{2}u_{x}}{\partial x^{2}}+\frac{\partial^{2}u_{x}}{\partial y^{2}}\right)+\;\frac{1}{1-2\nu }G\frac{\partial }{\partial x}\;\left(\frac{\partial u_{x}}{\partial x}+\frac{\partial u_{y}}{\partial y}\right)=0,\;G\left(\frac{\partial^{2}u_{y}}{\partial x^{2}}+\frac{\partial^{2}u_{y}}{\partial y^{2}}\right)+\;\frac{1}{1-2\nu }G\frac{\partial }{\partial y}\;\left(\frac{\partial u_{x}}{\partial x}+\frac{\partial u_{y}}{\partial y}\right)=0.$$

The most common definition of the engineering Poisson’s ratio (PR) is based on the assumption of small deformation. PR is simply defined as a negative ratio of the transverse to longitudinal strains. More generally the Poisson’s ratio for the longitudinal direction *l* and the transverse direction *t* can be written [41]

(10) $$\nu_{lt}=-\frac{\varepsilon_{t}}{\varepsilon_{l}}$$

where $\varepsilon_{t}$ and $\varepsilon_{l}$ are strains in transverse and longitudinal direction, respectively.

In the case of non-homogeneous material, the homogenization technique [42] is used. The effective value of the Poisson’s ratio is defined as a negative ratio of the average transverse to longitudinal strains:

(11) $$\nu_{eff}=-\frac{\langle \varepsilon_{t}\rangle }{\langle \varepsilon_{l}\rangle },$$

where $\langle \varepsilon_{t}\rangle$ and $\langle \varepsilon_{l}\rangle$ are average strains in transverse and longitudinal direction, respectively.

In the case of a large deformation, however, the expression describing effective PR might require more complex, nonlinear form. The logarithmic PR model is expressed by the following formula:

(12) $$\nu_{eff}=-\frac{\log\left(1+\langle \varepsilon_{t}\rangle \right)}{\log\left(1+\langle \varepsilon_{l}\rangle \right)},$$

but other models could also be considered [43].

The average strain is defined as $\langle \varepsilon \rangle =\int_{V}\varepsilon dV/V,$ where ε is strain in a given direction and *V* is the volume of the considered composite. This equation is proper only if volume *V* is fully filled by materials. If the analyzed composite is not fully filled, the strain is expressed as the change in length ΔL per unit of the original length L of the sample. When the sample is stretched or compressed along the *y*-axis, the average transverse strain is defined as:

(13) $$\langle \varepsilon_{t}\rangle \;=\frac{\int_{\Gamma_{1}}u_{x}d\Gamma }{L\int_{\Gamma_{1}}d\Gamma },$$

where $\Gamma_{1}$ is the free boundary (x=L). The average longitudinal strain is defined as:

(14) $$\langle \varepsilon_{l}\rangle \;=\frac{\int_{\Gamma_{2}}u_{y}d\Gamma }{L\int_{\Gamma_{2}}d\Gamma },$$

where $\Gamma_{2}$ is the boundary (y=L) where prescribed displacement boundary condition is applied.

The Poisson’s ratio of an isotropic, linearly elastic material can neither be less than −1.0 nor greater than $\frac{1}{D-1}$, where *D* is the number of dimensions of problem (*D* = 2 or 3). The limits of PR for isotropic solids possess fundamental significance. Shape is preserved at the lower limit of ν=−1. Volume is preserved at the upper limit ν=1/2 (for 3D) while area is preserved at upper limit ν=1 (for 2D). In anisotropic material, there is no bounds for the value of Poisson’s ratio [44].

## 3. Modelling of Thermoauxetic Composites

**Figure 1:**
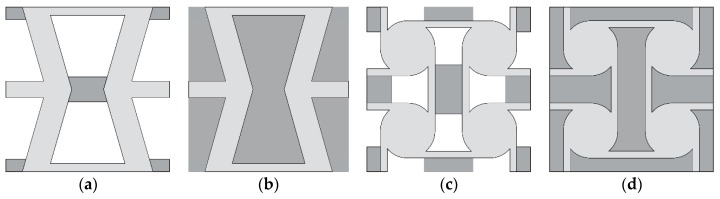
Analyzed auxetic unit cells (light gray) with linking/filling material (dark gray): (**a**) modified re-entrant honeycomb; (**b**) filled re-entrant honeycomb; (**c**) modified anti-tetrachiral; (**d**) filled anti-tetrachiral.

Four new structures built on the basis of two well-known auxetic structures, re-entrant honeycomb (RH) and anti-tetrachiral (AT), are analyzed in this paper. Both geometries have been modified so that empty spaces occurring in original structures are partially or completely filled with material characterized with material properties other than the material of the structure. The basic geometries are known to be auxetic so that negative values of the Poisson’s ratio of these structures is not surprising. For that reason, they were selected—to show that their well-known feature (auxeticity) could be modified and controlled through some structural modifications and thermal conditions. The temperature range is selected so that it could be easily attainable in experimental studies, the Young’s modulus of the stiffer material does not change significantly and the softer material remains in the range of elasticity. Hence, we obtained a tunable microstructure of auxetic/non-auxetic material. In Figure 1a, the RH structure with a link connecting re-entrant sides of the honeycomb cell is presented. The same geometry with completely filled voids is presented in Figure 1b. Anti-tetrachiral structure with linking elements is presented in Figure 1c and the same one with filled voids is presented in Figure 1d. In all considered cases, basic auxetic structures are made of stiff material whose material parameters are only slightly temperature-dependent, whereas linking (or filling) materials are characterized by Young’s modulus whose values change with temperature (from 200 K to 400 K) by at least one order of magnitude.

Modified RH geometry is characterized by several parameters; most of them describe auxetic geometry which has already been studied by many and which is not considered in this paper. The only parameter that defines exclusively the size of the linking element is *P*, therefore only this parameter is considered as a variable and its influence on the composite is studied. Similarly, in the case of AT geometry, the only parameter independent of auxetic structure geometry is F which also describes the size of the linking element. Hence, this is the only variable parameter analyzed for this geometry.

Boundary conditions (BC) applied to the analyzed quarter of structure are defined as follows:

- right boundary: x=L and y∈〈0,L〉 -free BC,
- left boundary: x=0 and y∈〈0,L〉 roller (symmetry) BC: n · u=0, where n is the normal unit vector to boundary,
- bottom boundary: y=0 and x∈〈0,L〉-roller (symmetry) BC: n · u=0,
- top boundary: y=L and x∈〈0,L〉-prescribed displacement: $u_{0}=(0,\Delta L_{y})$.

The is the prescribed displacement applied on the top boundary and it describes the extension during stretching or reduction during compressing in *y*-direction. In all considered cases $\Delta L_{y}=-0.1$ so all samples are compressed.

The behavior of considered samples depended on temperature, so thermal stresses were also initially taken into account. Several simulations, however, confirmed that the influence of thermal stresses is not crucial for the deformation mechanism under assumed thermal conditions, so this factor was omitted and the focus was only on the considered phenomenon. Moreover, a perfect interface is considered between both constituent materials so that the continuity of displacement field is assumed. However, in the case of experimental research one must carefully choose both materials as the differences in thermal expansion coefficients as well as other factors could influence the behavior of the considered structure as well as the values of resultant effective properties.

The analysis was performed with the use of material data available in Comsol Multiphysics material. The material data in this library is based on the experimental data obtained for selected materials [45,46,47,48]. For each considered case, both constituent materials of the considered composite structure were carefully chosen in order to provide required Young’s moduli ratio between them. Furthermore, the material used to create cellular auxetic structure had to be stiff and its mechanical properties were expected to not change significantly within assumed temperature range. Therefore steel, aluminum and Armco materials were selected as the values of Young’s moduli of these materials are either close to constant or changes slightly in a linear way. On the contrary, the value of Young’s modulus of the linking/filling material was expected to be considerably lower and strongly temperature-dependent so that polymeric materials (PMMA, polyamide) were selected. In order to present wider range of possible change in the effective PR and due to limited access to experimental data, computational material was also used, for which the Young’s modulus was assumed to be linearly changing according to the following formula: E(T) = (E_max_ − E_min_)(T_max_ − T)/(T_max_ − T_min_), where T_min_ and T_max_ defined the temperature range (T_min_ = 200 K and T_max_ = 400 K) and E_max_, E_min_ were respectively the maximum and the minimum value of Young’s moduli for these temperatures. The formula for E(T) is linear as nonlinearities would not change the result qualitatively but only quantitatively. Moreover, Young’s moduli of considered real materials are also changing quasi-linearly in the assumed temperature range. This computational material, however, allowed for better analysis and highlighting of the phenomenon. The value of Poisson’s ratio of the computational materials was assumed constant as stated in Table 1. Moreover, the use of this computational material allowed application of a wider range of Young’s modulus for assumed temperature range and resulted in a wider range of effective Poisson’s ratio of considered structures.

**Figure 2:**
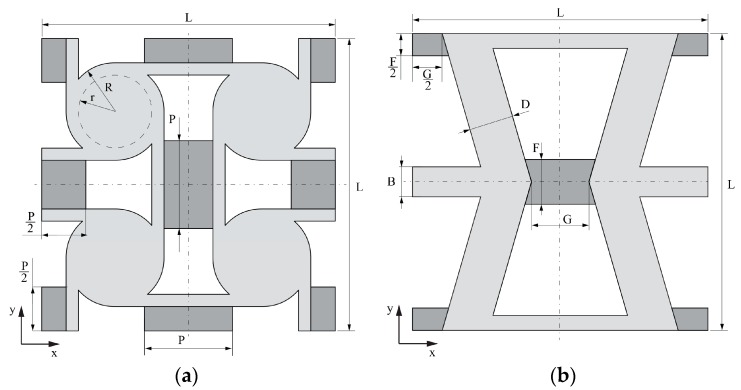
Geometries of analyzed unit cells: (**a**) modified anti-tetrachiral and (**b**) modified re-entrant honeycomb. Additional elements of unit cells in dark gray.

The geometries of both considered shapes are presented in the Figure 2.

## 4. Results

Finite element analysis was performed with the use of Comsol Multiphysics software. In all cases, the two-dimensional model was considered with the assumption of plane stress approximation. Triangular elements with 2nd order Lagrange polynomials as shape functions were used to create the mesh. The number of mesh elements varied and was in the range of 10,000–15,000 elements and about 150,000 degrees of freedom.

The results obtained for auxetic geometry with linking material were parametrized in order to present the influence of the geometrical parameter that defined basic dimension of linking elements. Hence, the plots consisted of multiple curves as opposed to plots obtained for geometries completely filled.

**Figure 3:**
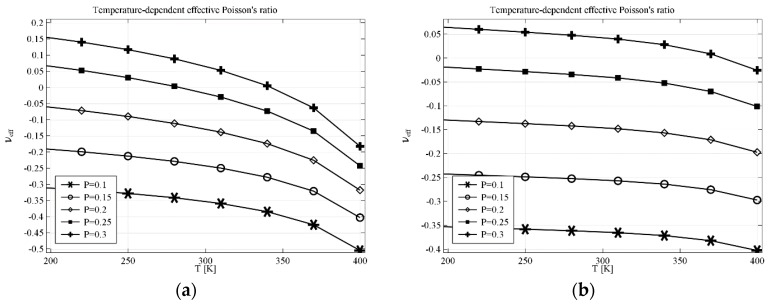
Effective Poisson’s ratio for AT (anti-tetrachiral) geometry with linking elements in the case of (**a**) computational linking material; (**b**) real linking material. Geometry parameters: *L* = 1, *R* = 0.33, *r* = 0.25, *P*—a variable parameter.

The results obtained for anti-tetrachiral geometry are presented in Figure 3a for the structure built with a linking element made of computational material and in Figure 3b for the structure made of real materials. The parameter *P* describes one dimension of linking a rectangular element (see Figure 2), the other dimension results from the geometry of the cell. The temperature dependence of effective Poisson’s ratio *υ_eff_* in the first case changes more than 0.3, e.g., for *P* = 0.3, *υ_eff_* changes from 0.15 at 200 K to almost −0.2 at 400 K. With the decrease of *P* value (thinning of the linking element), both values of *υ_eff_* and the range of *υ_eff_* decrease. The structure made of real materials shows the same tendency—the value of *υ_eff_* decreases with the increase of temperature. In particular, for *P* = 0.3, the value of *υ_eff_* changes form the positive value 0.07 at 200 K to −0.03 at 400 K.

**Figure 4:**
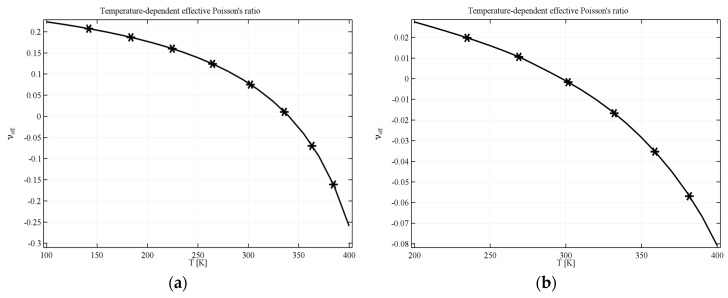
Effective Poisson’s ratio for AT geometry with filled voids in the case of (**a**) computational filling material; (**b**) real filling material. Parameters of the geometry: *L* = 1, *R* = 0.33, *r* = 0.25.

In the case of anti-tetrachiral geometry with completely filled voids, the resultant composite can still exhibit auxetic behavior if the ratio of Young’s moduli of the constituents is sufficient. Figure 4a shows the results obtained for a composite filled with computational material where the value of *υ_eff_* decreases from 0.2 at 200 K to −0.27 at 400 K. The results obtained for the structure made of real materials are different when it comes to the range of the effective Poisson’s ratio but the behavior is similar to that obtained with the computational material. The range of *υ_eff_* starts at 0.02 and decreases to −0.08 (see Figure 4b).

In general, the influence of temperature is non-linear and it strongly depends on the characteristics of each constituent material as well as on the geometry. It is, however, easily noticeable that in the case of geometry with linking elements, the relation between *υ_eff_* and temperature is almost linear in a wide range of temperatures. The same linear relation can be observed with the completely filled geometry, although the range is not so wide. This observation suggests that these characteristics can be optimized by careful selection of geometrical parameters and materials used.

**Figure 5:**
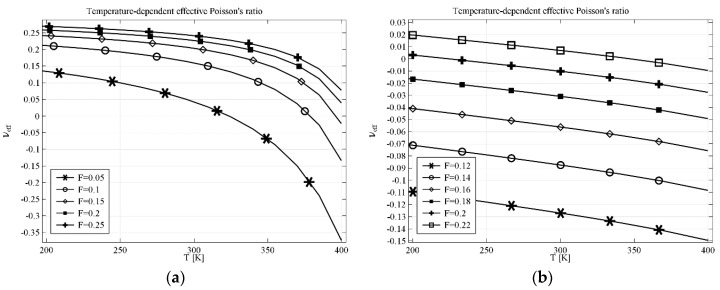
Effective Poisson’s ratio for RH (re-entrant honeycomb) geometry with linking elements in the case of (**a**) computational linking material; (**b**) real linking material. Parameters of the geometry: *L* = 1, *B* = 0.1, *D* = 0.15, *G* = 0.25, *F*—variable parameter.

Similar analysis was performed for the re-entrant honeycomb geometry. In the case of geometry modified by a linking element, parameter *F* is crucial. The results indicating both the impact of the temperature and the parameter *F* are presented in Figure 5a for computational element and in Figure 5b for real materials. Huge difference in behavior can be observed between the composite built with computational material and the one made of real materials. Yet, the difference is mainly due to the strong non-linear behavior in the range of temperatures above 350 K, presented in Figure 5a, while *υ_eff_* changes almost linearly in the whole range of temperatures in the case of structure made of real materials. This confirms that precise selection of materials and parameters is crucial. The results presented in Figure 5a show that, theoretically, it is possible to build a composite whose effective Poisson’s ratio is temperature tunable in a very wide range—in this case of *υ_eff_* starts form almost 0.15 at 200 K and decreases to −0.35 at 400 K for the parameter *F* = 0.05.

**Figure 6:**
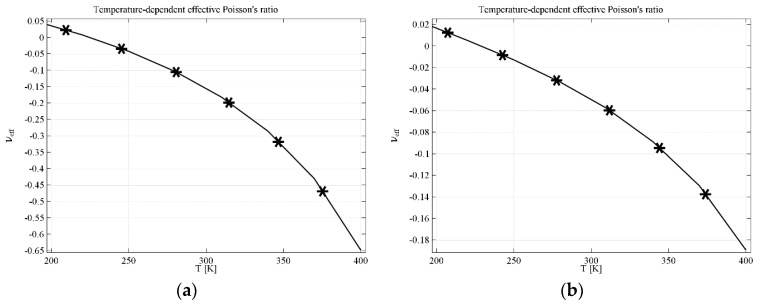
Effective Poisson’s ratio for RH geometry with filled voids in the case of (**a**) computational filling material; (**b**) real filling material. Parameters of the geometry: *L* = 1, *B* = 0.1, *D* = 0.15, *G* = 0.25, *F* = 0.16.

The last analysis deals with the AT geometry with completely filled voids. The results presented for computational filling material (see Figure 6a) and real filling material (see Figure 6b) may seem very similar, however *υ_eff_* ranges are very different. The value of *υ_eff_* decreases from 0.05 at 200 K to −0.65 at 400 K for the structure with computational material and such range is a huge change of effective Poisson’s ratio. Nonetheless, the range of *υ_eff_* for the structures made of real materials is also very wide: *υ_eff_* changes from 0.02 at 200 K to −0.18 at 400 K, which is very promising. It also confirms the possibility of manufacturing composite structures with tunable Poisson’s ratio controlled by temperature or other phenomena that could influence significantly the values of Young’s modulus.

**Figure 7:**
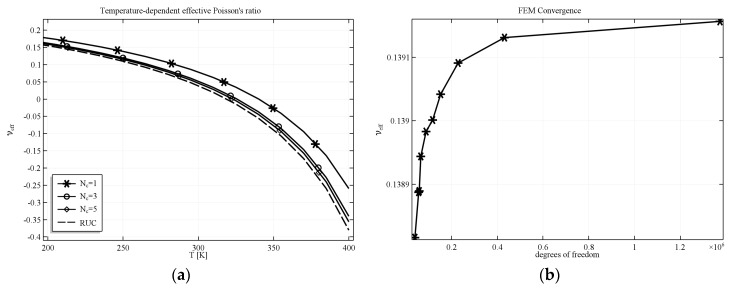
Effective Poisson’s ratio for AT geometry with filled voids in the case of computational filling material. (**a**) Convergence of the homogenized structure *Nc*—number of simulated unit cells in one quarter of the composite sample, RUC—repeating unit cell with periodic boundary conditions; (**b**) Result of convergence analysis with respect to the number of degrees of freedom for selected value of T = 250 K.

All simulations were performed with the use of boundary conditions mentioned above which means that the structure analyzed was in fact a quarter of material sample built of 4 unit cells. The main reason was that such complicated structures are difficult to be manufactured and so it could be easier to produce structures built of a small number of unit cell and compare them with the results of numerical analysis. However, the results were also validated for samples built of greater number of unit cells as well as with periodic structure. It has been shown [49] that results obtained for increasing number of repeating unit cell should converge to the results obtained for unit cell with periodic boundary conditions applied. Such convergence was also noticeable in our studies and exemplary results for selected numbers of unit cells *Nc* and repeating unit cell (RUC) are presented in Figure 7a. The dashed line represents the value of the effective PR computed for RUC with periodic boundary conditions. The convergence of FEM was also checked for increasing number of degrees of freedom. The plot showing the convergence of results with the increase of the number of degrees of freedom for selected value of temperature T = 250 K is presented in Figure 7b. It is easily noticeable that the method converges very well and the resultant effective Poisson’s ratio varies in a very small range (less than 1%).

## 5. Conclusions

The analysis of four composite structures is presented in this paper. Geometries are based on two popular auxetic structures, anti-tetrachiral and re-entrant honeycomb and modified by introduction of another material highly sensitive to the change of temperature. Resultant composite structures are also strongly temperature-dependent and so it is possible to actively tune the effective Poisson’s ratio and switch its value form positive to negative. The change of the effective Poisson’s ratio is determined by geometrical parameters of the structure as well as by temperature-dependent properties of each constituent. Results of this study were obtained numerically for materials subjected to temperatures in the range from 200 K to 400 K. All simulations were performed with the use of both computational materials, characterized by assumed elastic properties and real materials (aluminum, Armco iron, PMMA, polyamide) for reference. The results agreed well with each other and confirmed the possibility of manufacturing composite structures with tunable Poisson’s ratio controlled by temperature or other phenomena that could influence significantly the values of Young’s modulus. Naturally, computational material provided a wider range of effective Poisson’s ratio but the results obtained for real materials data were also very promising.

## Figures and Tables

**Table 1:** Basic characteristics of materials used in selected structures.

Geometry Type	Sample Materials *Material* 1/*Material* 2	Young’s Modulus, E [GPa] (200 K–400 K)		Poisson’s Ratio, *υ* (200 K–400 K)	
		Stiff Auxetic Structure Material 1	Linking/Filling Material 2	Stiff Auxetic Structure Material 1	Linking/Filling Material 2
Modified RH	steel/computational	200	200–20	0.33	0.33
	aluminum/polyamide	70	8.5–7.2	0.33	0.36
Filled RH	steel/computational	200	10–1	0.33	0.33
	Armco iron/PMMA	216–208	6.9–4.03	0.29	0.33–0.36
Modified AT	steel/computational	200	50–5	0.33	0.33
	aluminum/PMMA	70	8.5–7.2	0.33	0.33–0.36
Filled AT	steel/computational	200	20–2	0.33	0.33
	Armco iron/PMMA	216–208	6.9–4.03	0.29	0.33–0.36
